# Editorial for Special Issue “Molecular Research in Osteoarthritis and Osteoarticular Diseases”

**DOI:** 10.3390/cimb47050320

**Published:** 2025-04-30

**Authors:** Ye Liu

**Affiliations:** Department of Cell Biology, Van Andel Institute, Grand Rapids, MI 49503, USA; ye.liu@vai.org

## 1. Introduction

Osteoarthritis is a prevalent and debilitating disorder, affecting over 500 million people, primarily affecting aging adults [1]. It occurs when joint cartilage deteriorates, leading to pain, stiffness, and reduced mobility, which progressively worsen over time. Unlike other musculoskeletal tissues, such as bone, cartilage has a limited capacity for self-repair due to its lack of blood supply and resident stem cells. Currently, no disease-modifying osteoarthritis drugs are available; treatment options are limited to pain management and joint replacement, the latter being both costly and invasive. Given this unmet need, investigating the underlying mechanisms of osteoarthritis and the role of aging in its progression is crucial. Advancing our understanding of these processes could uncover new therapeutic opportunities to slow disease progression and promote cartilage repair.

## 2. An Overview of Published Articles

Recent studies featured in this Special Issue, “Molecular Research in Osteoarthritis and Osteoarticular Diseases”, have provided valuable insights into the molecular and cellular mechanisms underlying osteoarthritis.

Dijana Perković et al. (contribution 1) examined the effects of biological disease-modifying antirheumatic drugs (bDMARDs) on serum uric acid (SUA) levels in psoriatic arthritis (PsA). Their analysis of 87 patients treated with IL-17 inhibitors (secukinumab, ixekizumab) and TNFα inhibitors (golimumab, infliximab, adalimumab, certolizumab pegol, etanercept) between 2007 and 2021 revealed that 69% experienced reduced SUA levels, with IL-17 inhibitors showing the most significant reductions. Golimumab demonstrated the largest decrease. These findings suggest that bDMARDs, particularly IL-17 inhibitors, effectively lower SUA in PsA patients, warranting further research on the most potent treatment options.

Paloma Kênia de Moraes Berenguel Lossavaro et al. (contribution 2) investigated the antiarthritic and antinociceptive effects of ylang-ylang (Cananga odorata) essential oil (YEO) in experimental models. Their study showed that YEO treatment at 100 and 200 mg/kg doses reduced leukocyte infiltration and joint edema in zymosan-induced arthritis in mice. At the 200 mg/kg dose, YEO also decreased mechanical hyperalgesia, interleukin-6 (IL-6) levels, and cartilage destruction, demonstrating its anti-inflammatory properties. Additionally, YEO exhibited anti-hyperalgesic and antinociceptive effects in models of persistent inflammation and nociception, highlighting its potential as a therapeutic agent for arthritis and pain management.

Hua Yang et al. (contribution 3) investigated the safety of long-term exposure to a 0.2 T, 4 Hz rotating magnetic field (RMF) in mice. Over a 10-month period, the researchers assessed the health of two-month-old female C57BL/6 mice exposed to RMF and compared them to a SHAM group. They recorded body weights, conducted behavioral tests, and evaluated blood parameters, organ histology, and skeletal health. No adverse effects on body weight, behavior, organ morphology, or skeletal health were observed. While subtle changes in hormone and lipid levels were noted, they were not statistically significant. The study concluded that prolonged exposure to RMF did not cause chronic toxicity in mice, suggesting its potential for clinical application as a physical therapy.

Chiara Coppola et al. (contribution 4) provided a comprehensive review of osteoarthritis (OA), emphasizing its diagnosis, pathophysiology, and treatment strategies, with a particular focus on the therapeutic potential of natural extracts.

Kajetan Kiełbowski et al. (contribution 5) reviewed the role of alarmins in the pathogenesis of rheumatoid arthritis, osteoarthritis, and psoriasis. Alarmins, immune-activating factors released after cellular injury, play a crucial role in inducing inflammatory responses. The review discusses how altered alarmin levels contribute to the progression of these diseases and their potential as therapeutic targets. Additionally, the authors explore the impact of pharmacological treatments on the expression of alarmins, highlighting their significance in managing these inflammatory conditions.

Georgian-Longin Iacobescu et al. (contribution 6) explored the molecular basis of knee joint biomechanics in their review. They focused on genetic determinants that influence key biomechanical features such as ligament elasticity, meniscal resilience, and cartilage health. Special attention was given to collagen genes like COL1A1 and COL5A1 and their role in ligament strength and injury susceptibility. The review also addressed the genetic factors involved in knee osteoarthritis progression and the potential for personalized rehabilitation strategies, offering implications for injury prevention, treatment optimization, and regenerative medicine.

Theodora Adamantidi et al. (contribution 7) provided a comprehensive review on the inflammatory link between rheumatoid arthritis and thrombosis. While focusing on rheumatoid arthritis, the authors also include discussions on other conditions, such as osteoarthritis, highlighting how various types of arthritis are classified into two categories: non-inflammatory arthritis (like osteoarthritis) and inflammatory arthritis. The review explores how arthritis can be triggered by crystal deposition disorders, bacterial or viral infections (e.g., Staphylococcus aureus, Neisseria gonorrhea, Lyme disease complications, Parvovirus, Enterovirus, Hepatitis B (HBV), and Hepatitis E (HEV)), or autoimmune processes. It emphasizes the relationship between inflammation and thrombosis in these conditions.

## 3. Conclusions

Osteoarthritis remains a significant health burden, with limited treatment options available. However, recent molecular research has provided valuable insights into disease mechanisms, offering potential targets for future therapies. Continued investigation into inflammation, biomechanics, oxidative stress, epigenetics, and the gut–joint axis will be essential to developing effective disease-modifying interventions. We hope that the research presented in this Special Issue will inspire further exploration and innovation in the field of osteoarthritis.
